# Oxidative Stress and Protein Quality Control Systems in the Aged Canine Brain as a Model for Human Neurodegenerative Disorders

**DOI:** 10.1155/2015/940131

**Published:** 2015-05-11

**Authors:** Mariarita Romanucci, Leonardo Della Salda

**Affiliations:** Faculty of Veterinary Medicine, University of Teramo, 64100 Teramo, Italy

## Abstract

Aged dogs are considered the most suitable spontaneous animal model for studying normal aging and neurodegenerative diseases. Elderly canines naturally develop cognitive dysfunction and neuropathological hallmarks similar to those seen in humans, especially Alzheimer's disease-like pathology. Pet dogs also share similar living conditions and diets to humans. Oxidative damage accumulates in the canine brain during aging, making dogs a valid model for translational antioxidant treatment/prevention studies. Evidence suggests the presence of detective protein quality control systems, involving ubiquitin-proteasome system (UPS) and Heat Shock Proteins (HSPs), in the aged canine brain. Further studies on the canine model are needed to clarify the role of age-related changes in UPS activity and HSP expression in neurodegeneration in order to design novel treatment strategies, such as HSP-based therapies, aimed at improving chaperone defences against proteotoxic stress affecting brain during aging.

## 1. Introduction

The aged dogs naturally develop cognitive dysfunction and neuropathological hallmarks similar to those seen in normal elderly humans or in patients suffering from neurodegenerative conditions, particularly, Alzheimer's disease (AD). As well, they exhibit human-like individual variability in the aging process [1, 2].

Similar neuropathological changes include reduced brain volume with cortical/hippocampal atrophy, neuronal loss, and impaired neurogenesis [3–7]. Canines and humans display beta-amyloid (A*β*)-containing lesions with identical amino acid sequence [8, 9] and similar region specific progression of A*β* accumulation [1, 2, 8, 10–16]. In addition, canine A*β* peptides may undergo the same posttranslational modifications as occurring in humans [17, 18], making dogs a spontaneous aging model without the need for genetic modification or overexpression of mutant human proteins [2, 8]. The amount of A*β* plaque deposition in the dog brain is variable between individual animals, but it is related to the severity of cognitive decline [19–22].

Canine A*β* is ultrastructurally fibrillar and it generally aggregates into diffuse plaques [11, 23–26], mostly resembling early AD pathology [27–29]. Thus, the early AD-like canine neuropathological disease indicates that the dog is a valid model for prevention studies aimed at identifying AD therapeutics to be applied earlier in the disease progression in order to have a greater effect [2, 30].

Not only does canine and human A*β* exist in fibrillar conformation, but it may also be seen in a smaller, more soluble, oligomeric form, which is more toxic to synaptic and neuronal function and can be found in plaques [31–33]. Higher levels of A*β* oligomers are observable in both humans and canines with increasing cognitive decline [34, 35].

Human-like cerebrovascular abnormalities, particularly the cerebral amyloid angiopathy (CAA), are also frequently observed in aged dogs [11, 12, 23, 36–41], with a cerebral distribution similar to that seen in the human brain [42]. CAA is involved in cognitive decline in both humans [43–46] and canines [1, 11, 37, 38].

Thus, old dogs are considered a useful animal model for studying normal brain aging and neurodegenerative diseases, especially AD [1–3]. In particular, a comparative analysis of the changes described in the brains of selected elderly domestic animals and nonhuman primates indicated that the dog is the best natural animal model for further studies and observations on aging [47].

Pet dogs provide the unique advantage to share similar living conditions and diets to humans [2, 48, 49]. Canine cooperativeness also eliminates several physiological stressors that can affect cognitive testing results in other animal models such as rodents [2, 50].

Several drugs, especially statins, which have been proposed as novel therapeutics for AD, exhibit similar pharmacokinetics when administered to dogs or humans [51, 52]. Therefore, the canine model may be useful for the development of preventive or therapeutic interventions aimed at improving aged brain functions, which can be translated into human clinical trials [2].

Aging and age-related neurodegenerative disorders are usually associated with oxidative stress as one of the most important pathogenetic mechanisms contributing to neuronal dysfunction, degeneration and death, and cognitive decline in both humans and animals. A*β* accumulation may induce oxidative damage; at the same time, oxidative damage may contribute to A*β* deposition [2, 53–55]. Oxidative stress is one of the most common insults encountered by cells and it increases with age due to an excessive production of reactive oxygen species (ROS) or their derivatives. Mitochondria constitute the main source of these oxidants [56]. In normal conditions, adequate production and levels of endogenous antioxidants and antioxidant enzymes, quenching or metabolizing ROS, may reestablish a homeostatic balance. However, ROS overproduction associated with a progressive fail of protective mechanisms over time may result in oxidative damage to proteins, lipids, and/or DNA/RNA [1, 2]. In particular, neurodegenerative diseases are considered disorders of protein misfolding, so that they are usually referred to as* proteinopathies* [57–59]. Proteinopathies are characterized by the formation of fibrillar, amyloid-like structures with an elevated content of proteinaceous *β*-sheets [59–61]. Oligomers and protofibrils formed during protein aggregation have been demonstrated to be potent neurotoxins [59, 61, 62].

Besides antioxidants and antioxidant enzymes, multiple intracellular systems exist to protect cells from the proteotoxic stress, mainly represented by a network of chaperone and cochaperone proteins aimed at preventing protein misfolding and aggregation, and promote refolding of damaged proteins [59]. The posttranslational process that involves folding of newly synthesized proteins, as well as refolding or degradation of misfolded proteins, is termed* protein quality control* [63]. This chaperone network is mainly constituted by* Heat Shock Proteins* (HSPs) family members, which are one of the most evolutionarily conserved classes of molecules playing a fundamental role in the maintenance of cellular homeostasis, under both physiological and stress conditions, by acting as molecular chaperones in “protein holding” and “protein folding.” The higher levels of HSPs observed in tissues of longer-lived mammals and birds suggest that one mechanism underlying the evolution of longevity may be an improved protein homeostasis through an increased constitutive expression of HSPs [64].

**Figure 1:**
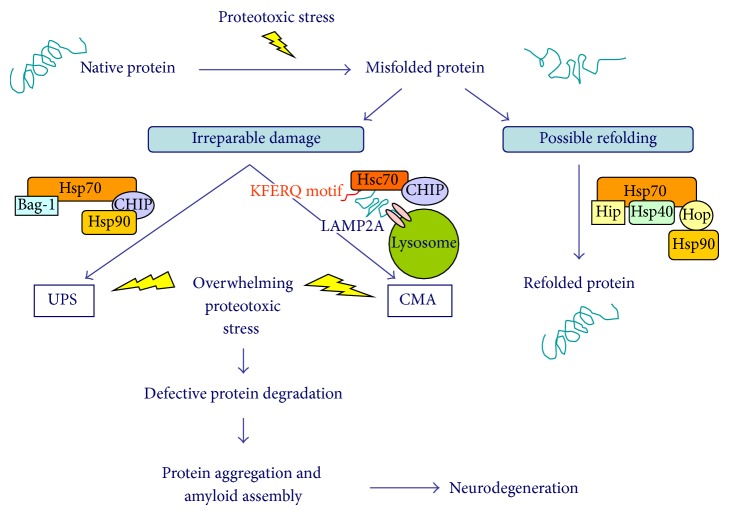
Intracellular protein quality control systems fighting against proteotoxic stress to avoid disruption of cellular functions by unfolded proteins. Under normal conditions, misfolded proteins can induce Hsp70 gene expression in order to be either refolded to native conformation or targeted for degradation if they are damaged beyond repair. The main cytosolic protein degradation pathway is represented by the ubiquitin-proteasome system (UPS). During protein degradation, both Hsp70 and Hsp90 bind to the cochaperone CHIP (carboxyl terminus of Hsp70-interacting protein), which serves as an E3 ubiquitin ligase by attaching a polyubiquitin chain to the irreparably damaged protein so that it can be targeted for proteasomal degradation. The proteasomal degradation process also requires the binding of BAG-1 (Bcl-2-associated athanogene) to the ATPase, N-terminal domain of Hsp70. Alternatively, CHIP may target misfolded proteins with a KFERQ motif for chaperone-mediated autophagy (CMA) by binding the heat shock cognate 70 (Hsc70 or Hsp73), which then guides the damaged proteins into the lysosome through the lysosomal-associated membrane transporter (LAMP2A). On the other hand, if proteins can be refolded into their native shape, BAG-1 binding to Hsp70 is blocked by the cochaperone Hip (Hsp70-interacting protein), whereas CHIP binding is blocked by the cochaperone Hop (Hsp70/90-organizing protein). Hsp40 and Hsp90 also bind to this protein refolding complex, promoting an ATP-dependent folding activity. Under conditions of overwhelming proteotoxic stress and defective protein degradation machineries, misfolded, damaged proteins may dramatically accumulate, aggregate, and kill cells.

When severely damaged proteins cannot be refolded into their native shapes, they can be targeted by chaperones to the primary cytosolic protein degradation pathway, the* ubiquitin-proteasome system* (UPS). In fact, the proteolytic destruction of abnormal proteins is usually performed by the proteasome, a large macromolecular complex that recognizes polyubiquitinated, damaged proteins. Alternatively, misfolded proteins with a KFERQ-related motif can be guided by HSPs (particularly, the heat shock cognate 70: Hsc70 or Hsp73) into the lysosome via translocation through the lysosomal-associated membrane transporter (LAMP2A), a process known as* chaperone-mediated autophagy* (CMA) [59, 65–67]. However, during aging, these systems may exhibit a reduced effectiveness and may be overwhelmed by the proteotoxic stress [59, 68, 69] (Figure 1). In particular, alterations in the proteasome activity may occur during aging and in several neurodegenerative conditions, possibly contributing to elevated intracellular levels of protein oxidation, protein aggregation, and consequent neurodegeneration [70, 71]. At the same time, glycated, oxidized, and aggregated proteins may inhibit the proteasome function [67].

Altered (increased or reduced) expression of many HSPs has been observed in the brain tissue of aged humans and animals as well as in tissues from elderly patients with neurodegeneration, indicating their involvement in the pathophysiology of aging and neurodegenerative disorders [59, 68] and making them potential targets for therapeutic interventions in aging and aging-related diseases [72, 73].

## 2. Oxidative Stress in the Aged Canine Brain

Oxidative injury can be measured by the amount of protein oxidation (carbonyl groups), by the end products of lipid peroxidation, including 4-hydroxynonenal, lipofuscin, lipofuscin-like pigments, and malondialdehyde, or by 8-hydroxy-2-deoxyguanosine (8OHdG) detecting oxidative damage to DNA/RNA [1, 2].

The aged canine brain experiences accumulation of carbonyl groups [74, 75], as well as increased lipid peroxidation [74, 76–79] and increased 8OHdG [29, 78]. Accumulation of carbonyl groups is associated with reduced endogenous antioxidant enzyme activity or protein levels, including glutamine synthetase and superoxide dismutase (SOD) [74, 79–81]. Increased oxidative damage to proteins and lipids correlates with cognitive dysfunctions in dogs [75, 77, 78]. Thus, aged dogs exhibit oxidative stress, similarly to humans with age-related neuropathological conditions, particularly AD [55, 82–88]. Since the canine brain shows an age-associated oxidative damage that correlates with an increased cognitive decline, aged dogs are considered a suitable model for translational antioxidant treatment/prevention studies [2].

### 2.1. Ubiquitin-Proteasome System (UPS) and Chaperones in Protein Quality Control in the Canine Model of Brain Aging

An increasing body of evidence indicates a decline in UPS and CMA activity during aging [69–71]. As well, increases or decreases of HSPs expression in aging and in neuropathological conditions have been observed in both humans and rodent models, with the responses depending on the different HSPs, disease, cell type, or brain region considered. Higher levels of some HSPs may represent a compensatory mechanism to reestablish homeostasis and slow down the progression of age-related disorders. However, such increases may be insufficient to counteract the overwhelming proteotoxic stress, since the levels and activity of several other HSPs and endogenous protective systems are reduced [59]. Understanding the roles played by the different HSPs in protein aggregation and subsequent neurotoxicity may lead to novel treatment strategies for aging and age-related proteinopathies directed to improve chaperone defences and reestablish the correct fate of misfolded proteins [68, 73].

UPS activity and HSP expression in the aged canine brain have not been extensively studied. However, some interesting data indicate the presence of an age-related decline of the basal expression of several components of the protein quality control systems in the canine hippocampus, a region playing an essential role in cognition and memory. In particular, an age-related decline of Psmd4, Psmb8, CHIP (carboxyl terminus of Hsp70-interacting protein), and egr1 expression, associated with an increase of Psmb9 and Hsp90 expression, suggests an age-related impairment of UPS activity [89]. In this respect, an age-related increase in density of ubiquitinated bodies has been found in the canine brain [3, 90], indicating a decreased proteolytic rate of damaged proteins. Egr1 is an inducible transcription factor with a confirmed role in synaptic plasticity [91] and regulation of the proteasome activity [92]. Many of the identified target genes of egr1 in neurons encode either components of the proteasome or proteins associated with ubiquitination and the intracellular protein degradation machinery [93]. Psma5, Psmb8, and Psmb9 are three proteasome egr1 target genes, which displayed heterogeneous modifications in aged dogs. In particular, the basal expression of Psma5, a gene encoding the *α*5-subunit of the 20S core particle of the proteasome, was not affected by aging. Conversely, downregulation of Psmb8 and upregulation of Psmb9 genes (which encode the catalytic subunits Lmp7 and Lmp2 of the 20S particle, resp.) were observed in the aged canine hippocampus [89]. The enhanced regulation of Psmb9 was consistent with the transcriptional suppression activity exerted by egr1 on Psmb9 [92]. The transcriptional activity of Psmd4 gene encoding the ubiquitin receptor located in the 19S regulatory particle of the proteasome (Rpn10) also showed an age-related reduction in the aged canine hippocampus [89].

The decline of UPS activity during aging in the canine brain was also indicated by an age-related reduction of the CHIP gene transcription associated with a decrease in CHIP protein levels in the canine hippocampus [89]. CHIP gene encodes a cochaperone protein with ubiquitin-ligase activity that has a crucial role in the UPS system [94, 95], and its deficiency has been shown to induce a decline in the proteasomal activity and an accelerated cellular senescence [96]. Since CHIP may also target proteins for CMA [67], its deficiency may be supposed to impair not only proteasomal but also lysosomal degradation, although alterations of Hsc70 expression were not observed in the aged canine brain [89]. On the other hand, an upregulation of Hsp90 was detected in the aged canine hippocampus [89], suggesting that the higher levels of Hsp90 observable with aging may be due to a higher load of damaged proteins. In this respect, enhanced levels of Hsp90 were observed in the 20S proteasomal fraction of aged mouse brain and liver, suggesting that the Hsp90 association with the 20S proteasome may be involved in rescuing cells from an age-related loss of proteasomal activity [97].

Differently from humans, canine neuropathological changes do not include the neurofibrillary tangle (NFT) formation [9, 98]. NFT formation is initiated by the polymerization of hyperphosphorylated tau into paired helical filaments (PHFs) and binding of ubiquitin. Tau is a microtubule-associated protein that stabilizes microtubules for axonal transport [47]. Although no study to date has observed NFTs in the brain of aged dogs, the increased phosphorylation detected at some tau sites in AD cases also occurs in cognitively impaired canines [76, 99–101]. Colocalization of p-tau and ubiquitin has also been observed in neurons of aged dogs with cognitive dysfunction and p-tau Ser396 is associated with canine cognitive decline [98]. Ubiquitin incorporation into protein aggregates is a consistent feature of several major human neurodegenerative disorders [102], including AD, and it is due to an impaired proteasomal degradation [103]. Whether UPS dysfunction is causally related to the disease pathogenesis or alternatively occurs as a result of the pathological condition remains to be elucidated [102].

The lack of NFT pathology in the canine brain could be due to differences in the tau protein sequence between canines and humans [47]. Whereas, in human AD brains, there are many sites of tau phosphorylation (including Ser189, 194, 202, 205, 207, 262, 396, 404, and Thr231) that contribute to PHF aggregation leading to NFT formation; tau phosphorylation in the brain of aged dogs with cognitive dysfunction may be limited to particular phosphorylation sites (i.e., Ser189, 207, and 396), so NFTs would not develop [98]. In addition, since NFTs are constituted by abnormally folded protein aggregates, the formation of such misfolded proteins could require a certain time scale, suggesting that in order to induce NFT formation in the canine brain, it would be necessary to lengthen the lifespan of the dog [104].

Notwithstanding this, an advantage to dogs not accumulating NFTs is that they may provide a selective valuable model for understanding the pathogenesis of A*β* pathology, especially early AD, as well as for preclinical testing of therapeutic approaches that specifically target this toxic protein [2, 98].

### 2.2. Antioxidant Treatments in the Canine Model of Normal Human Brain Aging and Neurodegenerative Disorders

Evidence suggests that the use of antioxidants results in reduction of oxidative damage and improvement of cognitive function in the canine model of human brain aging [105]. Several studies aimed at developing treatments for cognitive dysfunction in aged dogs were based on an antioxidant-rich diet in combination with a behavioural enrichment, including physical exercise, environmental and social enrichment, and cognitive training, which led to significant cognitive and neurobiological benefits [81, 106–108]. In particular, a significant increase in the enzymatic activities of Cu/Zn SOD, total SOD, and glutathione-S-transferase, as well as in the protein levels of heme oxygenase 1 (HO-1 or Hsp32), was observed in enriched environment-antioxidant-fortified food treated dogs [81]. In addition, aging dogs treated with human dose atorvastatin showed an upregulation of HO-1 in the parietal cortex, which exhibited significant negative correlations with oxidative stress indices and positive correlations with glutathione levels [109]. Hsp32 differs from the other HSPs, since it is more directly involved in antioxidant defence [59]. In response to oxidative stress, HO-1 induction may protect cells by promoting the catabolism of prooxidant metalloporphyrins, such as heme, to bile pigments (biliverdin and bilirubin) with free radical scavenging properties. However, since heme-derived free iron and carbon monoxide may increase the intracellular oxidative stress and mediate injury to mitochondrial membranes, a controversy was raised as to whether HO-1 upregulation observed in neurodegenerative diseases may exert a cytoprotective function or it may be an agent for further neurotoxicity [110]. Notwithstanding this, other studies have highlighted the protective functions of HO-1, also speculating that it might be a novel therapeutic target for neuroprotection. A more complete knowledge on the involvement of HO-1 in the pathogenesis of neuropathological diseases will be essential to successfully develop such promising therapeutic strategy [111, 112].

## 3. Conclusions

Old dogs are considered the best spontaneous animal model for studying normal brain aging and neurodegenerative diseases. The aged canines naturally develop cognitive dysfunction and neuropathological hallmarks similar to those seen in humans, especially AD-like pathology. Pet dogs also share similar living conditions and diets to humans. The canine brain shows age-associated oxidative lesions that correlate with increased cognitive decline, making dogs a suitable model for translational antioxidant treatment/prevention studies [1, 2]. Available data also indicate the presence of defective protein quality control systems in the aged canine brain [89]. Even though a HSP-based therapy appears to be a promising strategy for the treatment of diseases characterized by oxidative damage, there are still some major problems that must be overcome before this approach can be successfully applied. In particular, it is essential to understand how to safely and successfully upregulate HSPs at the right time and in the right location (specific cell type and brain region) [59]. An effective HSP-based therapy also needs to ensure that all the binding cochaperones are also present at the proper levels. If these difficulties could be overcome, an effective HSP-based therapy would be of great benefit to a variety of pathological conditions, including neurodegeneration, in which oxidative stress and protein misfolding play a critical role [113]. Further studies on the canine model will be useful to clarify the implications of the age-related changes in UPS activity and HSP expression in neurodegeneration in order to design novel treatment strategies targeting misfolded proteins.
